# Long-Term Impact of Optimum Contribution Selection Strategies on Local Livestock Breeds with Historical Introgression Using the Example of German Angler Cattle

**DOI:** 10.1534/g3.117.300272

**Published:** 2017-10-31

**Authors:** Yu Wang, Dierck Segelke, Reiner Emmerling, Jörn Bennewitz, Robin Wellmann

**Affiliations:** * Institute of Animal Science, University of Hohenheim, 70593 Stuttgart, Germany; † Vereinigte Informationssysteme Tierhaltung w.V., 27283 Verden, Germany; ‡ Institute of Animal Breeding, Bavarian State Research Centre for Agriculture, 85586 Grub, Germany

**Keywords:** optimum contribution selection, conservation, genetic gain, migrant contribution, runs of homozygosity

## Abstract

The long-term performance of different selection strategies was evaluated via simulation using the example of a local cattle breed, German Angler cattle. Different optimum contribution selection (OCS) approaches to maximize genetic gain were compared to a reference scenario without selection and truncation selection. The kinships and migrant contribution (MC) were estimated from genomic data. Truncation selection achieved the highest genetic gain but decreased diversity considerably at native alleles. It also caused the highest increase in MCs. Traditional OCS, which only constrains kinship, achieved almost the same genetic gain but also caused a small increase of MC and remarkably reduced the diversity of native alleles. When MC was required not to increase and the increase of kinship at native alleles was restricted, the MC levels and the diversity at native alleles were well managed, and the genetic gain was only slightly reduced. However, genetic progress was substantially lower in the scenario that aimed to recover the original genetic background. Truncation selection and traditional OCS selection both reduce the genetic originality of breeds with historical introgression. The inclusion of MC and kinship at native alleles as additional constraints in OCS showed great potential for conservation. Recovery of the original genetic background is possible but requires many generations of selection and reduces the genetic progress in performance traits. Hence, constraining MCs at their current values can be recommended to avoid further reduction of genetic originality.

Crossbreeding can have positive and negative consequences for managed livestock populations. The introgressive hybridization of breeds with high economic value is common to improve performance. Furthermore, the gene flow between populations can counteract the loss of genetic diversity and avoid inbreeding depression. However, it is possible that persistent introgression of genetic material causes breeds to become genetically extinct. For the management of local breeds with historical introgression, three conflicts have to be addressed, *i.e.*, the conflict between increasing genetic gain while managing the inbreeding level, the conflict between maintaining genetic diversity while controlling the loss of genetic uniqueness, and the conflict between increasing genetic gain while recovering the original genetic background. The traditional approach of OCS (traditional OCS) provides a solution to solve the first problem. It aims to maximize genetic gain while controlling the rate of inbreeding by optimizing the genetic contribution of each selection candidate to the next generation (Meuwissen 1997; Grundy *et al.* 1998; Woolliams *et al.* 2015). However, traditional OCS cannot solve the other problems. For breeds with historical introgression, although OCS is efficient for controlling the level of kinship and maintaining genetic diversity (Eynard *et al.* 2016), this diversity may be caused by a large proportion of genetic contributions from other breeds. Additionally, although OCS is efficient in increasing genetic gain, this genetic gain may be achieved by sustained introgression with high-yielding breeds. The reduction of genetic uniqueness due to high MCs and the reduction of diversity at native alleles is a risk for the conservation of the genetic background of the breed. Apart from focusing on high breeding values, reducing MCs to recover genetic originality could also be included as a breeding objective. Advanced OCS approaches could effectively maintain native allele diversity and genetic originality, while ensuring genetic improvement by including MC and kinship at native alleles (Wellmann *et al.* 2012) as additional constraints, which has been shown for OCS based on pedigree information (Wang *et al.* 2017).

High-density marker panels of single-nucleotide polymorphisms (SNPs) allow us to obtain more accurate estimates of kinship than pedigrees, as it is common for pedigrees to contain errors (Ron *et al.* 1996). In addition, genotype-based kinship reflects the actual relatedness between two individuals, whereas pedigree-based estimates are only expectations (Visscher *et al.* 2006). Furthermore, genotype-based kinship is able to capture the relationships due to distant common ancestors that pedigree-based estimates fail to reflect. Thus, using genotype-based kinship is more efficient than using a pedigree-based approach for the conservation of local breeds (Toro *et al.* 2014; Mészáros *et al.* 2015), especially for the removal of undesirable genetic materials (Amador *et al.* 2013). Currently, there are three approaches to estimate kinships from genome-wide SNPs. The first one is the molecular kinship, *i.e.*, the proportion of SNPs that are identical by state (IBS) (Eding and Meuwissen 2001; Caballero and Toro 2002). The second one is the genomic covariance between individuals computed from gene contents (VanRaden 2008; Yang *et al.* 2010). The third one is the segment-based kinship computed from shared haplotype segments, which are also known as runs of homozygosity (Gusev *et al.* 2009; de Cara *et al.* 2013; Rodríguez-Ramilo *et al.* 2015; Gómez-Romano *et al.* 2016). Both molecular kinship and genomic relationship matrices have the disadvantage of being biased due to the preselection of markers included in the SNP panel (Nielsen 2000; McTavish and Hillis 2015). Moreover, increasing genetic diversity by reducing average molecular kinship drives allele frequencies toward 0.5 and increases the frequency of rare deleterious alleles. Thus, it accumulates deleterious variants in the genome and may reduce the fitness of the population (de Cara *et al.* 2011, 2013). The use of segment-based kinship has been shown to provide a good compromise between maintaining diversity and fitness levels in populations. The estimate based on segments reflects recent identity by descent rather than IBS (Keller *et al.* 2011). In this study, the segment-based kinship will be used in the optimization process.

Genomic information can also be used to estimate the breed composition of an individual (Frkonja *et al.* 2012). Software packages for predicting breed composition are usually based on either hidden Markov model clustering algorithms or maximum likelihood procedures (Pritchard *et al.* 2000; Alexander *et al.* 2009; Baran *et al.* 2012). Such analysis can be carried out by Admixture (Alexander *et al.* 2009) or Structure (Pritchard *et al.* 2000), where individuals are assumed to be unrelated, and linkage disequilibrium (LD) is not taken into account. Another approach is to assign haplotype segments to the breeds in which they have maximum frequency, which is carried out by *optiSel* (Wellmann 2017).

The objective of this study was to evaluate the long-term performance of different genomic OCS strategies, using the example of a local cattle breed, by simulating several subsequent generations. The scenarios were compared not only with respect to the genetic gain but also with respect to parameters measuring genetic diversity and genetic uniqueness.

## Methods

### Data

The dataset consisted of genotype information for 889 individuals belonging to five cattle breeds: 268 Angler, 200 Fleckvieh, 200 Holstein-Friesian, 200 Red Holstein, and 21 Norwegian Red. The targeted breed in this study was Angler cattle, which is a dual-purpose cattle breed with an emphasis on milk production. It is mainly located in the northern part of Germany (Bennewitz and Meuwissen 2005). The reference breeds, which include animals from the nontargeted breeds, were only used for the identification of native haplotype segments in Angler cattle. Two hundred Fleckvieh animals were genotyped with the Illumina BovineHD BeadChip (HD), and the remaining animals were genotyped with the Illumina BovineSNP50 BeadChip (50K) with standard quality control parameters. SNPs that were not available for all breeds were discarded. Finally, 23,448 autosomal SNPs were used for the analysis. Haplotypes were phased for all breeds jointly as part of a larger dataset, and missing genotypes were imputed using BEAGLE software (Browning and Browning 2007). To visualize the relationship between Angler cattle and the other four breeds, principal component analysis (PCA) (Price *et al.* 2006) was performed on the SNP genotypes using PLINK 1.9 software (Chang *et al.* 2015).

### Simulation

The simulations comprised two parts. First, a base population ($G_{0}$) was generated from the phased genotypes of the Angler cattle. Second, this base population was managed for the following 10 nonoverlapping generations in accordance with the respective scenario.

The base population $G_{0},$ consisting of 1000 simulated individuals, was generated from genotypes of 131 Angler bulls and 137 Angler cows based on a random sampling of gametes. The animals from other breeds, which were used to identifying native segments, remained the same for each generation. The selection process started from generation $G_{0}.$ For all scenarios, the optimum genetic contribution of each selection candidate (${\text{c}}_{\text{i}}$) to the next generation was calculated for each generation. The corresponding number of offspring of each parent was generated, and mates were allocated randomly. Offspring received haplotypes from their parents via Mendelian inheritance, allowing recombination to occur according to the length of the chromosomes; *i.e.*, one crossover occurs on average on a chromosome of size 1 M (Weng *et al.* 2014). For all generations, the population size remained 1000 (500 males and 500 females). For each scenario, five replicates were simulated and the results presented are averages over replicates.

A total of 1500 SNPs were sampled randomly without replacement to become quantitative trait loci (QTL). The QTL effects were sampled from a γ distribution with a shape parameter of 0.4 (Meuwissen *et al.* 2001) and standardized afterward. The effect of each QTL had a 50% chance of being positive or negative. The highest positive QTL effects were assigned to SNPs that were more frequent in the reference breeds than in Angler cattle. Hence, the mean breeding value in Angler cattle was lower than the mean breeding value in the reference breeds that were used for introgression.

The simulated true breeding value (TBV) of animal j was calculated as the sum of all QTL effects:

$${\text{TBV}}_{\text{j}}=\sum_{\text{k=1}}^{{\text{n}}_{\text{QTL}}}{\text{a}}_{\text{k}}\cdot Q_{\text{kj}},$$

where $n_{\text{QTL}}=1500$ is the number of QTL, $a_{k}$ is the additive effect of QTL k, and $Q_{\text{kj}}$ is the QTL genotype of individual j at locus k. The genotypes were coded as 0, 1, or 2, as the number of copies of the alternative allele. For each individual, an estimated breeding value (EBV) for total merit with the reliability of 0.75 was simulated as:

$${\text{EBV}}_{\text{j}}=\mu_{\text{EBV}}+r^{2}\left({\text{TBV}}_{\text{j}}-\mu_{\text{EBV}}\right)+E_{\text{j}}$$,

where $\mu_{\text{EBV}}$ is the mean of the breeding values of the corresponding generation, $E_{\text{j}}$ is a residual term sampled from a normal distribution with mean 0, and variance $\sigma_{\text{E}}^{2}=r^{2}\left(1-r^{2}\right)\sigma_{\text{TBV}}^{2}.$

### MC, kinships, and diversity parameters

For calculating the kinship matrices and MC, the origin of each marker had to be determined for each haplotype from the breed of interest. A haplotype was classified to be native in Angler cattle at a particular marker position if the frequency of the segment containing the marker was sufficiently low in all reference breeds. Only haplotype segments consisting of ≥ 20 consecutive markers and a minimum length of 2.5 Mb were considered. A marker was classified to be native in Angler cattle if the frequency of the segment containing the marker was < 0.01 in all reference breeds. The MC of each individual was calculated as the proportion of its genome that was not classified to be native. The mathematical definitions can be found in the Appendix. For identification of the origin of markers, the R package *optiSel* (Wellmann 2017) was used.

Two SNP-based kinship parameters were considered, which are denoted as $f_{\text{SEG}}$ and $f_{\text{SEG}|\text{N}}.$ Kinship $f_{\text{SEG}}$ between individual i and individual j (element of the matrix $f_{SEG}$) is the probability that two alleles taken from a random position from randomly chosen haplotypes of both individuals belong to a shared segment, which is in accordance with de Cara *et al.* (2013). The mean kinship $f_{SEG}$ for the offspring generation is estimated as $c^{’}f_{SEG}c,$ where c is the vector of optimum genetic contributions of all selection candidates. In addition, average kinships among different breeds were calculated from a segment-based kinship matrix that included individuals from all breeds.

For breeds with historical introgression, Wellmann *et al.* (2012) proposed that kinship at native alleles should be restricted to preserve local breeds. The kinship $f_{\text{SEG}|\text{N}}$ is the conditional probability that two alleles taken at random from the population belong to a shared segment, given that they are native. For the computation of the segment-based kinship $f_{\text{SEG}}$ and the kinship at native alleles $f_{\text{SEG}|\text{N}}$, we used R package *optiSel* (Wellmann 2017). The corresponding pedigree-based kinships were referred to as $f_{\text{A}}$ and $f_{\text{D}}$ in Wang *et al.* (2017). The mathematical definitions can be found in the Appendix.

Three additional genetic parameters were calculated to evaluate the level of genetic diversity of Angler cattle, *i.e.*, the average observed heterozygosity $\left(H_{\text{O}}\right),$ the variance of the TBVs ($\sigma_{\text{TBV}}^{2}$), and the genic variance ($\sigma_{\text{A}}^{2}$). The observed heterozygosity quantifies the amount of genetic variation due to polymorphic loci, which is an important parameter of estimating genetic variation within a population (Gregorius 1978). We calculated the $H_{\text{O}}$ of each generation in each scenario with software PLINK 1.9 (Chang *et al.* 2015). The genic variance was calculated as

$$\sigma_{\text{A}}^{2}=\sum_{m=1}^{{\text{n}}_{\text{QTL}}}2{\text{p}}_{\text{m}}\left(1-{\text{p}}_{\text{m}}\right){\text{a}}_{\text{m}}^{2}$$,

where $n_{\text{QTL}}=1500$ is the number of QTL, $p_{\text{m}}$is the allele frequency at locus m, and $a_{\text{m}}$ is the additive effect of QTL m (Falconer and Mackay 1996).

### Optimization scenarios

Except for the reference scenario, the objective of all the other scenarios was to maximize the genetic gain of the following generation, so the objective function was $c^{’}EBV,$ where EBV is a vector of the EBVs of all selection candidates. Three OCS scenarios were considered and compared to two non-OCS scenarios, *i.e.*, a reference scenario without selection and a truncation selection scenario.

#### Reference scenario (REF):

In this scenario, all animals were used as parents and each selection candidate had two offspring. Thus, no selection or optimization was done. The effective population size ($N_{\text{e}}$) is 2000, thus, the increase of kinship is negligible.

#### Truncation selection (TS):

Maintenance of an effective population size of 100 was envisaged, as recommended in Meuwissen (2009). Calculated from $1/{\text{N}}_{\text{e}}\approx 1/4{\text{N}}_{\text{Sire}}+1/4{\text{N}}_{\text{dam}}$ (Falconer and Mackay 1996), 26 bulls with the highest EBVs were selected for breeding to create the following generation by truncation selection. In this scenario, all selected bulls had equal contributions to the offspring. Note that the effective size in this scenario is expected to deviate slightly from 100 because the formula does not take into account how the individuals with highest breeding values are related.

#### Traditional OCS method (OCS-I):

To restrict the rate of inbreeding, the upper bound of kinship $f_{\text{SEG}}$ was defined as follows. Since the targeted effective population size was $N_{\text{e}}$=100, the desired rate of inbreeding, which can be calculated from $\Delta \text{F}=\;\text{1}/2{\text{N}}_{\text{e}}$ (Falconer and Mackay 1996), was 0.5% per generation. The threshold for $f_{\text{SEG}}$ of generation t+1 was calculated as:

$$\text{ub}.{\text{f}}_{\text{SEG}}{}_{\text{t}+1}=\bar{{\text{f}}_{\text{SEG}}{}_{\text{t}}}+\left(1-{\bar{{\text{f}}_{\text{SEG}}}}_{\text{t}}\right)\Delta \text{F},$$

where $\bar{f_{\text{SEG}}{}_{\text{t}}}$ is the average kinship of the population in generation t.

#### OCS with constraint on kinship ${\text{f}}_{\text{SEG}},$ kinship ${\text{f}}_{\text{SEG}|\text{N}}$, and MC (OCS-II):

The constraint of kinship $f_{\text{SEG}}$ was the same as in the OCS-I scenario. Additionally, constraints on conditional kinship $f_{\text{SEG}|\text{N}}$ and MC were applied. The upper bound threshold for $f_{\text{SEG}|\text{N}}$ in generation t+1 was calculated as:

$$\text{ub}.{\text{f}}_{\text{SEG}|\text{N}}{}_{\text{t}+1}=\bar{{\text{f}}_{\text{SEG}|\text{N}}{}_{\text{t}}}+\left(1-{\bar{{\text{f}}_{\text{SEG}|\text{N}}}}_{\text{t}}\right)\Delta \text{F}$$,

where $\bar{f_{\text{SEG}|{\text{N}}_{\text{t}}}}$ is the mean kinship at native alleles of the population at generation t. Additionally, we required that for each generation, the average level for the estimated MC did not exceed the average level in the base generation $G_{0}$ ($\bar{{\text{MC}}_{{\text{G}}_{0}}}$).

#### OCS with constraint on kinship ${\text{f}}_{\text{SEG}},$ kinship ${\text{f}}_{\text{SEG}|\text{N}}$, and reduced level of MC (OCS-III):

The upper bounds of kinship ${\text{f}}_{\text{SEG}}$ and of kinship ${\text{f}}_{\text{SEG}|\text{N}}$ were the same as in scenario OCS-II for each generation. Additionally, in this scenario, we required that the MC level estimated from haplotypes decreased by 3% per generation.

Several reasonable conditions were made for all scenarios. The genetic contribution of a selection candidate $\left({\text{c}}_{\text{i}}\right),$ expressed as the proportion of genetic material originating from this individual in the next generation, was assumed to be nonnegative $\left({\text{c}}_{\text{i}}\geq 0\right).$ In diploid species, each sex group contributes half of the genes to the gene pool. Thus, the sum of genetic contribution of all selection candidates of a sex was 0.5; *i.e.*, $c^{’}s=0.5\;\text{and}\;c^{’}d=0.5,$ where s and d are vectors for indicators of a candidate’s sex. For all OCS scenarios, optimization was done only for males. All females were assumed to have equal numbers of offspring. All 500 males were used as selection candidates, which reflects a breeding program with genomic selection in which a substantial number of the bull calves are genotyped.

The specific values for each constraint are shown in Supplemental Material, Table S1 in File S3. Solver “cccp” (Pfaff 2014), which was called from the R package *optiSel* version 0.9.1(Wellmann 2017), was used to solve the optimization problems. Five replicates per scenario were simulated and the results presented are averages across replicates.

### Data availability

The data used in this study are available as supplemental files. File S1 contains SNP ID numbers and locations. File S2 contains simulated genotypes for each individual of the Angler cattle base generation ${\text{G}}_{0}.$

## Results

### Analysis of base generation (${\text{G}}_{0}$)

**Figure 1:**
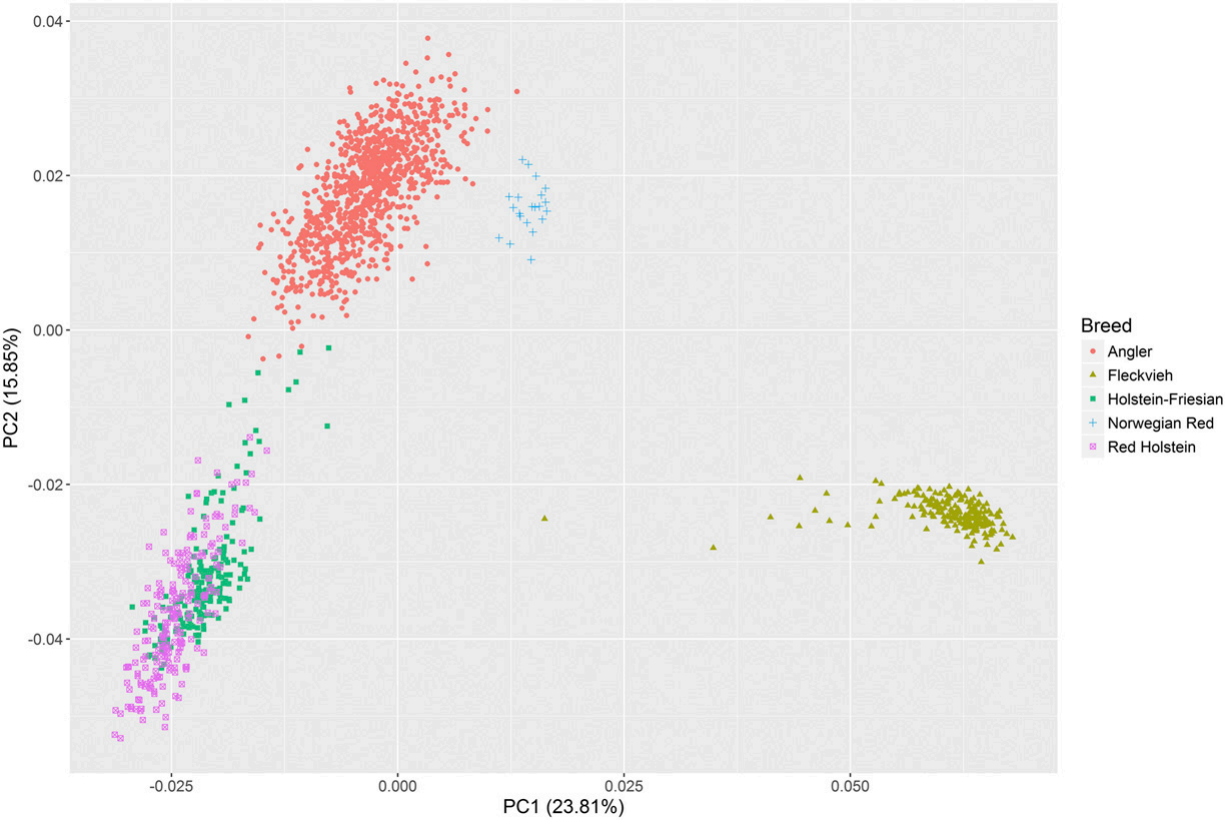
Plot of the first two principal components (PCs) for the dataset of the simulated base population $G_{0}.$ The analysis was based on 1621 individuals and 23,448 single-nucleotide polymorphisms. Different colors and shapes represent individuals from different breeds.

The simulated base generation reflects the structure of the genotyped animals well. PCA plots of both populations were almost identical (Figure 1 and Figure S1). The first and second principal components (PC1 and PC2) separated animals in the simulated base generation according to their breed (Figure 1). PC1 explained 23.81% of the total variance and distinguished Fleckvieh from the other four breeds. Angler was separated from the Holstein family by PC2, which explained 15.85% of the total variance. Overlap existed between Holstein-Friesian and Red Holstein since Red Holstein is known to be a subpopulation of Holstein-Friesian.

Relationships within and between breeds are shown in Table 1. The smallest average kinship within a breed was found in Angler (0.048). This shows that Angler has a higher genetic diversity and lower inbreeding than the other breeds. Angler had a close relationship with Red Holstein (0.039) and Holstein-Friesian (0.037), a moderately close relationship with Norwegian Red (0.017), and a distant relationship with Fleckvieh (0.004). This is in agreement with the estimated genetic contributions that the Angler breed has from other breeds, which were 0.448 from Holstein-Friesian and Red Holstein, 0.152 from Norwegian Red, and 0.021 from Fleckvieh (data not shown).

**Table 1:** Average kinship ($f_{SEG}$) among five breeds of the simulated base generation $G_{0}$ based on shared segments

	Angler	Red Holstein	Holstein-Friesian	Norwegian Red	Fleckvieh
Angler	0.048	0.039	0.037	0.017	0.004
Red Holstein		0.110	0.085	0.007	0.004
Holstein-Friesian			0.095	0.007	0.004
Norwegian Red				0.086	0.002
Fleckvieh					0.061

A basic statistical analysis of the simulated TBVs of animals based on each breed group is presented in Table 2. Angler and Fleckvieh had relatively low average TBVs, with a mean of 0.560 and 0.367, respectively. Holstein-Friesian, Norwegian Red, and Red Holstein had relatively high average TBVs, with means of 2.160, 2.390, and 2.431, respectively (as desired). As shown in Figure S2, there was a positive correlation between MC and TBV in the base population of Angler cattle.

**Table 2:** Basic statistics of the simulated true breeding values of each breed group of base generation $G_{0}$

Breed	*N*	Mean	SD
Angler	1000	0.560	0.444
Fleckvieh	200	0.367	0.275
Holstein-Friesian	200	2.160	0.275
Norwegian Red	200	2.390	0.270
Red Holstein	21	2.431	0.276

### Values of each parameter obtained in five scenarios

The mean and SD of the parameter values at the starting stage [base generation (${\text{G}}_{0}$)] and final stage [10th generation (${\text{G}}_{10}$)] for all scenarios are shown in Table 3. The mean and SD were estimated from five replicates. The values of the corresponding parameter for all generations can be found in Tables S2–S8 in File S3.

**Table 3:** Basic statistics of each parameter achieved in base generation $G_{0}$ and $G_{10}$ for each selection scenario

	Parameters*^a^*						
	EBV	MC	$f_{SEG}$	$f_{SEG|N}$	$H_{O}$	$\sigma_{TBV}^{2}$	$\sigma_{A}^{2}$
Beginning of selection (${\text{G}}_{0}$)							
	0.561 ± 0.000	0.622 ± 0.000	0.048 ± 0.000	0.061 ± 0.000	0.367 ± 0.000	0.197 ± 0.000	0.075 ± 0.000
End of selection (${\text{G}}_{10}$)							
REF	0.558 ± 0.020	0.587 ± 0.002	0.044 ± 0.001	0.048 ± 0.001	0.364 ± 0.001	0.091 ± 0.005	0.075 ± 0.001
TS	3.002 ± 0.062	0.679 ± 0.005	0.115 ± 0.005	0.157 ± 0.009	0.346 ± 0.002	0.044 ± 0.002	0.049 ± 0.001
OCS-I	2.915 ± 0.026	0.638 ± 0.008	0.094 ± 0.001	0.136 ± 0.004	0.351 ± 0.001	0.049 ± 0.002	0.052 ± 0.002
OCS-II	2.757 ± 0.059	0.617 ± 0.001	0.085 ± 0.001	0.104 ± 0.001	0.353 ± 0.001	0.056 ± 0.001	0.054 ± 0.002
OCS-III	1.825 ± 0.106	0.455 ± 0.001	0.073 ± 0.001	0.104 ± 0.001	0.355 ± 0.001	0.065 ± 0.003	0.063 ± 0.002

EBV, estimated breeding value; MC, migrant contribution; $f_{\text{SEG}},$ kinship; $f_{\text{SEG}|\text{N}},$ kinship at native alleles; $H_{\text{O}},$ average heterozygosity; $\sigma_{\text{TBV}}^{2},$ variance of true breeding value; $\sigma_{\text{A}}^{2},$ genic variance.

a Parameters estimated in each generation of each scenario.

### EBV and MC

**Figure 2:**
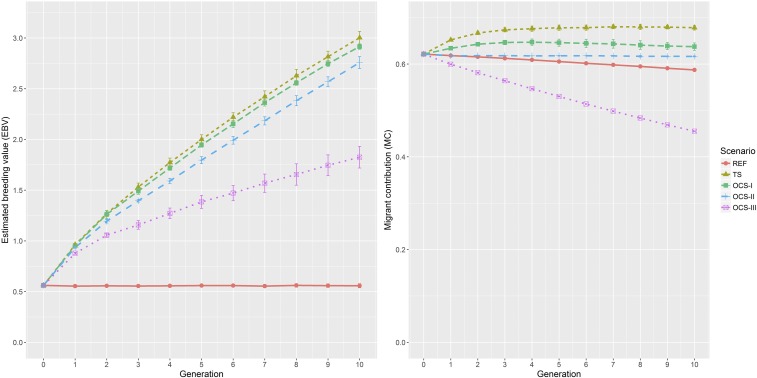
Average estimated breeding values (left) and MC (right) achieved in each generation of each selection scenario. $f_{\text{SEG}},$ kinship; $f_{\text{SEG}|\text{N}},$ kinship at native alleles; MC, migrant contribution; OCS, optimum contribution selection; OCS-I, traditional OCS method; OCS-II, OCS with constraint on kinship ${\text{f}}_{\text{SEG}},$ kinship ${\text{f}}_{\text{SEG}|\text{N}}$, and MC; OCS-III, OCS with constraint on kinship ${\text{f}}_{\text{SEG}},$ kinship ${\text{f}}_{\text{SEG}|\text{N}}$, and reduced level of MC; REF, reference scenario; TS, truncation selection.

Except for the reference scenario, the average EBV level of Angler cattle increased in varying degrees from generation to generation in all scenarios, which is shown in Figure 2 (left). The EBV level remained stable at ∼0.558 in REF. The average increase of EBV was ∼0.9 genic SD per generation in TS and OCS-I. The average EBV in ${\text{G}}_{10}$ was very similar in both scenarios TS and OCS-I. The genetic gain was lower in scenario OCS-II, which achieved an average EBV of 2.757 in ${\text{G}}_{10},$ and was considerably lower in scenario OCS-III, for which the mean EBV was 1.825 in ${\text{G}}_{10}.$

MC decreased slightly from 0.622 to 0.587 in the reference scenario because old introgressed haplotype segments were split by crossing-over into smaller pieces and could no longer be detected in ${\text{G}}_{10}.$ In contrast, with the increase of EBV in scenarios TS and OCS-I, the level of MC increased to a different extent (Figure 2, right). In scenario TS, MC increased from 0.622 in ${\text{G}}_{0}$ to 0.676 in ${\text{G}}_{4}$ and became stable in later generations. Similarly, in scenario OCS-I, MC increased to 0.647 at ${\text{G}}_{4}$ and became stable afterward. For scenarios OCS-II and OCS-III, MC was set as a constraint. Thus, the average MC values obtained in each generation were approximately equal to the threshold setting in the corresponding generation with a rather small SD; that is, the estimated MC remained 0.618 in OCS-II and decreased by 3% each generation in scenario OCS-III.

### Kinship $f_{SEG}$ and kinship $f_{SEG|N}$

**Figure 3:**
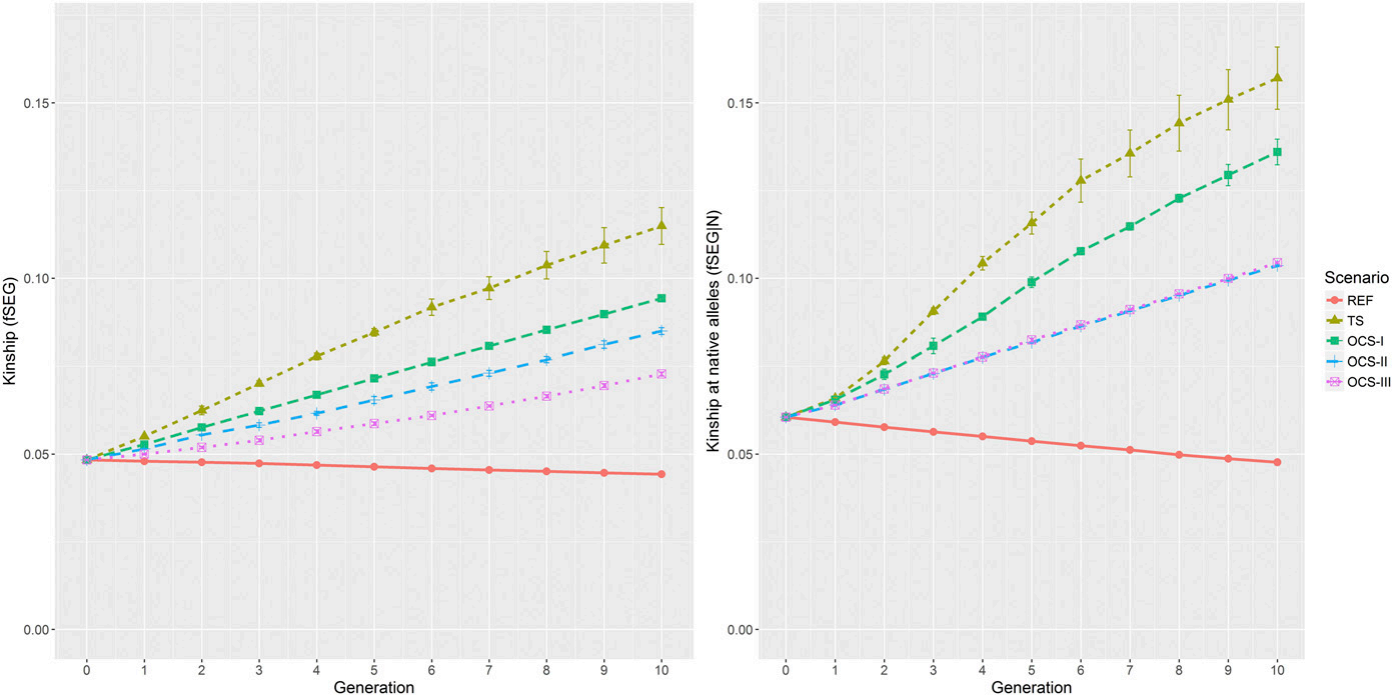
Average kinship $f_{\text{SEG}}$ (left) and kinship at native alleles $f_{\text{SEG}|\text{N}}$ (right) achieved in each generation of each selection scenario. $f_{\text{SEG}},$ kinship; $f_{\text{SEG}|\text{N}},$ kinship at native alleles; MC, migrant contribution; OCS, optimum contribution selection; OCS-I, traditional OCS method; OCS-II, OCS with constraint on kinship ${\text{f}}_{\text{SEG}},$ kinship ${\text{f}}_{\text{SEG}|\text{N}}$, and MC; OCS-III, OCS with constraint on kinship ${\text{f}}_{\text{SEG}},$ kinship ${\text{f}}_{\text{SEG}|\text{N}}$, and reduced level of MC; REF, reference scenario; TS, truncation selection.

Kinship ${\text{f}}_{\text{SEG}}$ and kinship${\text{ f}}_{\text{SEG}|\text{N}}$ increased from generation to generation to varying extents, except for scenario REF, which can be seen in Figure 3 (left:${\text{f}}_{\text{SEG}};$ right: ${\text{f}}_{\text{SEG}|\text{N}}$). Kinship ${\text{f}}_{\text{SEG}}$ had a small reduction in REF from 0.048 to 0.044, which was because old segments were split into smaller pieces, so after some generations, the pieces were no longer involved in shared segments. Kinship increased the most in scenario TS, which moved from 0.048 in ${\text{G}}_{0}$ to 0.115 in ${\text{G}}_{10}.$ Kinship ${\text{f}}_{\text{SEG}}$ was set as a constraint in the other three scenarios. For scenario OCS-I, the ${\text{f}}_{\text{SEG}}$ value of each generation equals the corresponding value of the constraint setting. For scenario OCS-II, in generation ${\text{G}}_{10},$ the .${\text{f}}_{\text{SEG}}.$ value increased to 0.085, which is lower than the constraint setting. The smallest mean kinship (0.073) was obtained for scenario OCS-III.

Estimated kinship ${\text{f}}_{\text{SEG}|\text{N}}$ decreased from 0.061 in ${\text{G}}_{0}$ to 0.048 in ${\text{G}}_{10}$ for the reference scenario because some old introgressed segments were split into small pieces by crossing-over, so the alleles included in the segments were classified as native and contributed to the estimated diversity at native alleles. Kinship ${\text{f}}_{\text{SEG}|\text{N}}$ increased faster in scenarios TS and OCS-I than kinship${\text{ f}}_{\text{SEG}}.$ The value increased from 0.061 in ${\text{G}}_{0}$ to 0.157 in TS and to 0.136 in OCS-I. For scenarios OCS-II and OCS-III, ${\text{f}}_{\text{SEG}|\text{N}}$ was set as a constraint parameter. In all generations of both scenarios, the ${\text{f}}_{\text{SEG}|\text{N}}$ values were equal to the corresponding constraint setting of $\text{ub}.{\text{f}}_{\text{SEG}|\text{N}},$ with an SD close to zero.

### Diversity parameters $\left(H_{O},\sigma_{TBV}^{2}\;and\;\sigma_{A}^{2}\right)\;$

**Figure 4:**
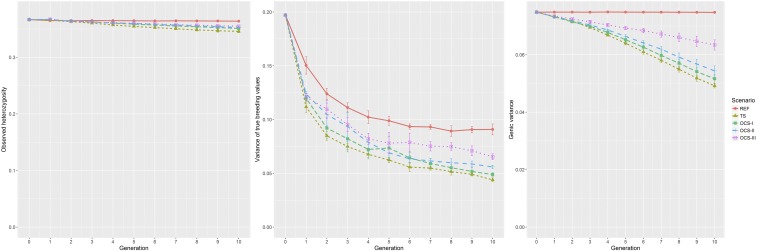
Average observed heterozygosity $H_{\text{O}}$ (left), variance of true breeding values $\sigma_{\text{TBV}}^{2}$ (middle), and genetic variance $\sigma_{\text{A}}^{2}$ (right) achieved in each generation of each selection scenario. $f_{\text{SEG}},$ kinship; $f_{\text{SEG}|\text{N}},$ kinship at native alleles; MC, migrant contribution; OCS, optimum contribution selection; OCS-I, traditional OCS method; OCS-II, OCS with constraint on kinship ${\text{f}}_{\text{SEG}},$ kinship ${\text{f}}_{\text{SEG}|\text{N}}$, and MC; OCS-III, OCS with constraint on kinship ${\text{f}}_{\text{SEG}},$ kinship ${\text{f}}_{\text{SEG}|\text{N}}$, and reduced level of MC; REF, reference scenario; TS, truncation selection.

The diversity parameters are shown in Figure 4 (left: $H_{\text{O}};$ middle: $\sigma_{\text{TBV}}^{2};$ and right: $\sigma_{\text{A}}^{2}$). As expected, all diversity values in $G_{10}$ of REF are higher than the corresponding values of all the other scenarios. The value of $H_{\text{O}}$ and $\sigma_{\text{A}}^{2}$ remained nearly unchanged from $G_{0}$ to $G_{10}.$ The value of $\sigma_{\text{TBV}}^{2}$ decreased considerably from 0.197 to 0.091 from $G_{0}$ to $G_{10},$ which is still higher than the level of all the other scenarios (see Table 3). $\sigma_{\text{TBV}}^{2}$ was larger than $\sigma_{\text{A}}^{2}$ in $G_{0},$ which was due to the effects caused by different chromosomes being correlated.

For all scenarios, $H_{\text{O}}$ is relatively stable compared to $\sigma_{\text{TBV}}^{2}$ and $\sigma_{\text{A}}^{2}.$ The greatest reduction of $H_{\text{O}}$ was found in scenario TS, which moved from 0.367 in $G_{0}$ to 0.346 in $G_{10}.$ A similar trend but a faster reduction showed the genic variance $\sigma_{\text{A}}^{2}.$ In scenario TS, the average $\sigma_{\text{A}}^{2}$ decreased from 0.075 in $G_{0}$ to 0.049 in $G_{10}.$ A higher $\sigma_{\text{A}}^{2}$ value in $G_{10}$ was achieved in OCS-I (0.052) and OCS-II (0.054), and the highest genic variance was maintained in OCS-III (0.063).

In all scenarios, the level of $\sigma_{\text{TBV}}^{2}$ decreased considerably from $G_{0}$ to $G_{1}.$ Thereafter, it decreased at a slower rate and approached the level of the genic variance around $G_{6}.$ For the scenarios with selection, the variance of TBVs in ${\text{G}}_{10}$ was very similar to the genic variance. It was 0.044 in TS, 0.049 in OCS-I, 0.056 in OCS-II, and the highest level was maintained in scenario OCS-III (0.065). In all scenarios, the average $\sigma_{\text{TBV}}^{2}$ level was much higher than the average genic variance $\sigma_{\text{A}}^{2}$ in the first generations. This can be seen in Figure S3 using the example of scenario OCS-II.

## Discussion

In this study, we evaluated the long-term performance of five different scenarios for maximizing genetic gain in the context of conserving breeds with historic introgression using the example of Angler cattle. MCs and kinships at native alleles based on shared haplotype segments were restricted in some scenarios. A large proportion of the Angler breed’s genetic background was contributed by other breeds, especially Holstein, which is in accordance with results obtained from pedigree records (Wang *et al.* 2017). Truncation selection achieved the highest genetic gain among the five scenarios with the highest degree of reduction in genetic diversity. Traditional OCS (OCS-I) achieved a slightly lower genetic gain and slightly higher genetic diversity compared to truncation selection. However, both are inappropriate for the situation of Angler cattle, as both reduced the diversity at native alleles considerably and increased the MC. Constraining MC and kinship at native alleles enabled recovery of the genetic originality but also slowed the genetic progress in performance traits compared to truncation selection and traditional OCS.

### Genetic progress *vs.* genetic conservation

Due to the protocol for simulating QTL effects, a positive correlation between MC and EBV was observed, which is in agreement with results obtained from the pedigree information (Wang *et al.* 2017). The positive correlation persisted in all generations in all five scenarios (data not shown). There was no genetic progress in REF due to the absence of selection. Truncation selection and traditional OCS achieved similar genetic gain. When MC and kinship at native alleles were constrained, the genetic gain in performance traits was reduced. Hence, to achieve maximum genetic gain, it is essential to allow for the introgression of foreign genetic material.

Maximizing genetic gain is not the only objective of a breeding program. To recover the genetic background of the original endangered population from admixtures, two goals must be set: maintain the genetic diversity at native alleles and remove the introgressed genomic material in the long run. The average MC of the population can be treated as a parameter for measuring genetic uniqueness. Among the five scenarios, truncation selection has the least ability to maintain genetic uniqueness. Although the situation improves in traditional OCS, the estimated MC level in ${\text{G}}_{10}$ is still higher than at the starting stage and higher than in the reference scenario without selection. This is in accordance with what we obtained from OCS based on pedigrees. The reason why traditional OCS did not cause a larger increase in MC is probably that most MCs were from related Holstein cattle. Thus, increasing genetic gain by increasing MCs would also increase the average kinship in Angler cattle, so restricting average kinship implicitly restricted the MC. Similarly, in Amador *et al.* (2011), traditional OCS did not eliminate any exogenous representation but kept the value constant, irrespective of the number of generations that had elapsed before management started. Genetic originality could be maintained with the OCS-II method, while genetic gain was only marginally lower, as in traditional OCS and truncation selection.

In OCS-III, the EBV level kept increasing throughout all generations, even though the original genetic background was gradually reconstructed and the highest diversities were maintained with this method. Compared to OCS-II, the reduced genetic progress in OCS-III is directly linked to the strictness of the constraint MC setting. Due to the conflict between achieving genetic gain and maintaining genetic uniqueness, a breeding organization should choose MC constraint settings carefully to achieve both breeding purposes.

In truncation selection, 26 sires with the highest breeding values were selected along with 500 dams to achieve an effective population size of 100 in each generation. However, the formula from which the number of selected sires was obtained did not take into account that the individuals with the highest breeding values were related because they had high genetic contributions from closely related Holstein ancestors. Thus, the rate of inbreeding in truncation selection was higher than in the OCS scenarios. Compared to truncation selection, traditional OCS has good performance in controlling inbreeding via restricting average relatedness in the offspring.

### Different kinship estimators

The predictions from ${\text{f}}_{\text{SEG}}$ and ${\text{f}}_{\text{SEG}|\text{N}}$ from *optiSel* were close to the values estimated from offspring haplotypes (results not shown). However, they were slightly larger because some segments were split by crossing-over into small pieces, so they did not contribute to the kinship estimated from offspring haplotypes. This indicates that the estimate obtained from offspring haplotypes is slightly biased. The rate of inbreeding estimated from segments remained 0.5% per generation in traditional OCS, in accordance with the constraint level setting.

Kinship ${\text{f}}_{\text{SEG}}$ estimates the probability that randomly chosen alleles are IBD. However, it lacks the ability to distinguish whether the alleles originated from native or migrant ancestors. In scenario OCS-I, where ${\text{f}}_{\text{SEG}}$ was restricted, the increasing rate of ${\text{f}}_{\text{SEG}|\text{N}}$ was higher than the increasing rate of ${\text{f}}_{\text{SEG}}.$ This suggests that restricting only ${\text{f}}_{\text{SEG}}$ had the consequence that diversity at introgressed segments was maintained, which tend to have higher breeding values. But a depletion of diversity at native segments could not be avoided. Because kinship and kinship at native alleles are correlated, restricting ${\text{f}}_{\text{SEG}|\text{N}}$ implicitly restricted ${\text{f}}_{\text{SEG}},$ so in scenarios OCS-II and OCS-III, the mean kinship ${\text{f}}_{\text{SEG}}$ was lower than the corresponding constraint setting. This suggests that the constraint for ${\text{f}}_{\text{SEG}}$ could be skipped if ${\text{f}}_{\text{SEG}|\text{N}}$ and MC are constrained. Similar results were obtained from pedigree information from Wang *et al.* (2017).

### MCs

In general, it must be distinguished whether MCs predominantly originate from closely related ancestors originating from a single high-yielding breed, or if different unrelated breeds have been used for upgrading. In the Angler breed, they predominantly originated from related Holstein ancestors, so reducing MC in OCS-III was meant to reduce the amount of genetic material contributed by Holstein cattle, which had a positive effect on the genetic diversity. Thus, the mean kinship ${\text{f}}_{\text{SEG}}$ in OCS-III was smaller than in all other scenarios.

### Criteria for detecting shared segments

It has been suggested that the marker density of the SNP chip used, the minimum length of the shared segment, the number of genotyping errors allowed, and the minimum number of SNPs allowed in a single shared segment are likely to remarkably influence kinship estimates based on shared segments (Peripolli *et al.* 2016). However, to date, there is a lack of consensus in establishing the criteria for determining these parameters, which makes it difficult to compare results from different studies. In this paper, the minimum number of markers in a segment was 20. A shorter minimum length for shared segments allows detection of more ancient inbreeding from common ancestors occurring many generations back (Curik *et al.* 2014), but it also increases the probability that segments that are identical by chance are considered to be IBD. The minimum length of a segment was 2.50 Mb because, in this case, the correlation between the contribution from the Holstein breed estimated from the pedigree and genotype was high (0.93, data not shown) and the genetic contribution from the Fleckvieh breed was low (∼0.02), in accordance with pedigree records. The average MC of the Angler population was 0.62, which is also similar to the average MC level obtained from the pedigree (Wang *et al.* 2017). If shorter segments were also to be used, then kinships of individuals would be affected more by very old common ancestors and would consequently be higher.

### Reduction of estimates in unselected populations

Estimated parameter values for MC, ${\text{f}}_{\text{SEG}}$, and ${\text{f}}_{\text{SEG}|\text{N}}$ decreased slightly from generation to generation in REF, even though there was no selection in this scenario. This reduction of the above three parameters was caused by recombination, which shortened the length of the haplotype segments (Stam 1980) until they became too short to meet the criteria for being segments, which led to the reduction of ${\text{f}}_{\text{SEG}}.$ Moreover, if recombination occurred near a particular marker position at an introgressed haplotype segment, then the segment containing the marker could no longer be detected in other breeds. Hence, the marker failed to meet the criteria of belonging to a foreign segment. This gave rise to the reduction of estimated MC. Moreover, since the marker was now classified as native, it contributed to the diversity at native alleles, which caused a reduction in ${\text{f}}_{\text{SEG}|\text{N}}.$ Consequently, the estimated MC should be compared with the estimates obtained from the reference scenario rather than with generation $G_{0}.$ In particular, in scenario OCS-II, in which the constraint for MC was set equal to the MC in generation $G_{0},$ only the estimate of MC was kept constant, whereas the true MC was effectively increased. There are two possibilities to avoid this increase. Either the constraint for MC in generation ${\text{G}}_{\text{t}}$ is set equal to the mean MC in generation ${\text{G}}_{\text{t}}$ (rather than the mean MC in generation $G_{0}$), or another method could be used to estimate the origins of the haplotype segments in generation ${\text{G}}_{\text{t}+1}.$ That is, the origin of a marker could be set equal to the origin of the marker in the parental haplotype from which it originates.

### Genetic diversity parameters

Different parameters can be used to measure genetic diversity, such as the percentage of polymorphic loci, the number of alleles per locus, expected heterozygosity, etc. (Harper and Hawksworth 1994). The genetic variation within a breed is of major importance for the conservation of local breeds. In addition to $f_{\text{SEG}}$ and ${\text{f}}_{\text{SEG}|\text{N}},$ three further parameters were considered for evaluation of the level of genetic diversity, *i.e.*, the average observed heterozygosity $\left({\text{H}}_{\text{O}}\right),$ the variance of TBV (${\text{σ}}_{\text{TBV}}^{2}$), and the genic variance ($\sigma_{\text{A}}^{2}$). Restricting kinship at native alleles and MCs not only had an impact on recovering the original genetic background, but also showed the most potential in conserving genetic diversity among all scenarios. In this study, a similar decreasing pattern of ${\text{H}}_{\text{O}}$ and $\sigma_{\text{A}}^{2}$ was observed, with a smaller extent of reduction for parameter ${\text{H}}_{\text{O}}.$ This is because ${\text{H}}_{\text{O}}$ is predominantly influenced by neutral alleles (Gregorius 1978).

The additive genetic variance $\sigma_{\text{TBV}}^{2}$ was substantially larger than the genic variance in the first generations and decreased to a large amount from generation ${\text{G}}_{0}$ to generation ${\text{G}}_{5}.$ This was predominantly because the genetic effects of different chromosomes were correlated in the Angler breed. The contribution of the covariance between different chromosomes to the variance of TBV was 0.089, so in the absence of the covariance, the variance of TBV should be 0.108. The Angler cattle in generation ${\text{G}}_{0}$ had different contributions from the high-yielding Holstein cattle. For an individual with a high contribution from Holstein cattle, the breeding values of all chromosomes tended to be high, whereas for an individual with a low contribution from Holstein, the breeding values of all chromosomes tended to be low. Consequently, in the first generations, there was covariance between effects of different chromosomes, which contributed to the variance of the breeding values. Additionally, the Bulmer Effect (Bulmer 1971) and the changes in LD due to selection (Bijma 2012; Gorjanc *et al.* 2015) contributed to the difference between $\sigma_{\text{TBV}}^{2}$ and $\sigma_{\text{A}}^{2}.$

### Conclusions

Advanced OCS strategies enable the achievement of a balance between the different breeding goals of populations with historic introgression, which are to improve the genetic progress, recover the original genetic background, and conserve genetic diversity. Here, truncation selection and traditional OCS achieved the highest genetic gain, but both reduced the genetic originality of the breed by depleting diversity at native alleles and increasing MCs. However, maintaining genetic originality is crucial for conserving breeds with historical introgression. The inclusion of MC and kinship at native alleles as additional constraints in OCS showed great potential for conservation. Recovering the original genetic background is possible but requires many generations of selection and reduces the genetic progress. Thus, it is essential to set an appropriate constraint for MC in order to balance both breeding goals, which are to achieve genetic progress and to recover the original genetic background of local breeds.

## Supplementary Material

Supplemental material is available online at www.g3journal.org/lookup/suppl/doi:10.1534/g3.117.300272/-/DC1.

## Appendix

### Native Segments

Each individual i has a maternal haplotype ${\text{H}}_{1\text{i}}$ and a paternal haplotype ${\text{H}}_{2\text{i}}.$ For haplotype ${\text{H}}_{\text{vi}}$ of Angler i, the frequency of the segment at marker m in the set of animals from reference breed B was calculated as:

$${\text{p}}_{\text{vi}}\left(\text{m};\text{B}\right)=\frac{1}{2{\text{*N}}_{\text{B}}}\sum_{\text{j}\in \text{B}}1_{\text{m}\in \text{S}\left({\text{H}}_{\text{vi}},{\text{H}}_{1\text{j}}\right)}+1_{\text{m}\in \text{S}\left({\text{H}}_{\text{vi}},{\text{H}}_{2\text{j}}\right)},$$

where $N_{\text{B}}$ is the number of individuals from breed B, set $\text{S}\left({\text{H}}_{\text{vi}},{\text{H}}_{\text{wj}}\right)$ contains all markers belonging to segments, which are identical in haplotypes $H_{\text{vi}}$ and ${\text{H}}_{\text{wj}},$ and $1_{\text{m}\in \text{S}\left({\text{H}}_{\text{vi}},{\text{H}}_{2\text{j}}\right)}=1$ if marker m belongs to such a segment.

A marker m from haplotype $H_{\text{vi}}$ was classified as native (${\text{N}}_{\text{vi}}\left(\text{m}\right)=1$) if the frequency of the segment containing marker m is smaller than ε=0.01 in all reference breeds. That is,

$${\text{N}}_{\text{vi}}\left(\text{m}\right)=1\Leftrightarrow \underset{\text{B}}{\max}{\text{p}}_{\text{vi}}\left(\text{m};\text{B}\right)<\mathrm{\varepsilon ,}$$

where the maximum was taken over all reference breeds, which were Fleckvieh, Holstein-Friesian, Red Holstein, and Norwegian Red in our study.

The native contribution $N_{\text{i}}$ of individual i is the proportion of the genome included in native segments. That is,

$${\text{N}}_{\text{i}}=\frac{1}{2\text{L}}\sum_{m}{\text{L}}_{\text{m}}\cdot \left({\text{N}}_{1\text{i}}\left(\text{m}\right)+{\text{N}}_{2\text{i}}\left(\text{m}\right)\right).$$

Here, $L_{\text{m}}$ is the length of the genome region in megabases represented by marker m, and L is the length of the genome in megabases. The MC of individual i is ${\text{MC}}_{\text{i}}=1-{\text{N}}_{\text{i}}.$

### Kinship $f_{SEG}$ and $f_{SEG|N}$

The kinship $f_{\text{SEG}}$ between individuals i and j (element of matrix $f_{SEG}$) is the probability that two alleles taken at random from individuals i and j belong to identical segments:

$${\text{f}}_{\text{SEG}}{}_{\text{ij}}=\frac{1}{4}\sum_{\text{v},\text{w}=1}^{2}\frac{\sum_{m}{\text{L}}_{\text{m}}\cdot 1_{\text{m}\in \text{S}\left({\text{H}}_{\text{vi}},{\text{H}}_{\text{wj}}\right)}}{\text{L}}\;,$$

where all parameters involved are the same as previously explained.

The kinship $f_{\text{SEG}|\text{N}}$ in the offspring is the conditional probability that two alleles taken at random from the offspring belong to identical segments, given that they are native. The mean kinship $f_{\text{SEG}|\text{N}}$ of the following generation is estimated as:

$${\text{f}}_{\text{SEG}|\text{N}}=\frac{c^{\prime }f_{SEG\&\text{N}}c}{c^{\prime }f_{N}c},$$

where $f_{SEG\&\text{N}}$ is a matrix containing the probabilities that two alleles taken at random from both individuals belong to identical segments and are native, and $f_{N}$ is a matrix containing the probabilities that two alleles taken at random from both individuals are native:

$${\text{f}}_{\text{SEG}\&\text{N}}\left(\text{i},\text{j}\right)=\frac{1}{4}\sum_{\text{v},\text{w}=1}^{2}\frac{\sum_{m}{\text{L}}_{\text{m}}\cdot {\text{N}}_{\text{vi}}\left(\text{m}\right)\cdot {\text{N}}_{\text{wj}}\left(\text{m}\right)\cdot 1_{\text{m}\in \text{S}\left({\text{H}}_{\text{vi}},{\text{H}}_{\text{wj}}\right)}}{L},$$

$${\text{f}}_{\text{N}}\left(\text{i},\text{j}\right)=\frac{1}{4}\sum_{\text{v},\text{w}=1}^{2}\frac{\sum_{m}{\text{L}}_{\text{m}}\cdot {\text{N}}_{\text{vi}}\left(\text{m}\right)\cdot {\text{N}}_{\text{wj}}\left(\text{m}\right)}{L}$$.
