# Supplementary figures and images for: Long-Term Impact of Optimum Contribution Selection Strategies on Local Livestock Breeds with Historical Introgression Using the Example of German Angler Cattle
Source: G3 (Bethesda). 2017 Oct 31;7(12):4009–18. doi: 10.1534/g3.117.300272 (PMC5714497; doi:10.1534/g3.117.300272)

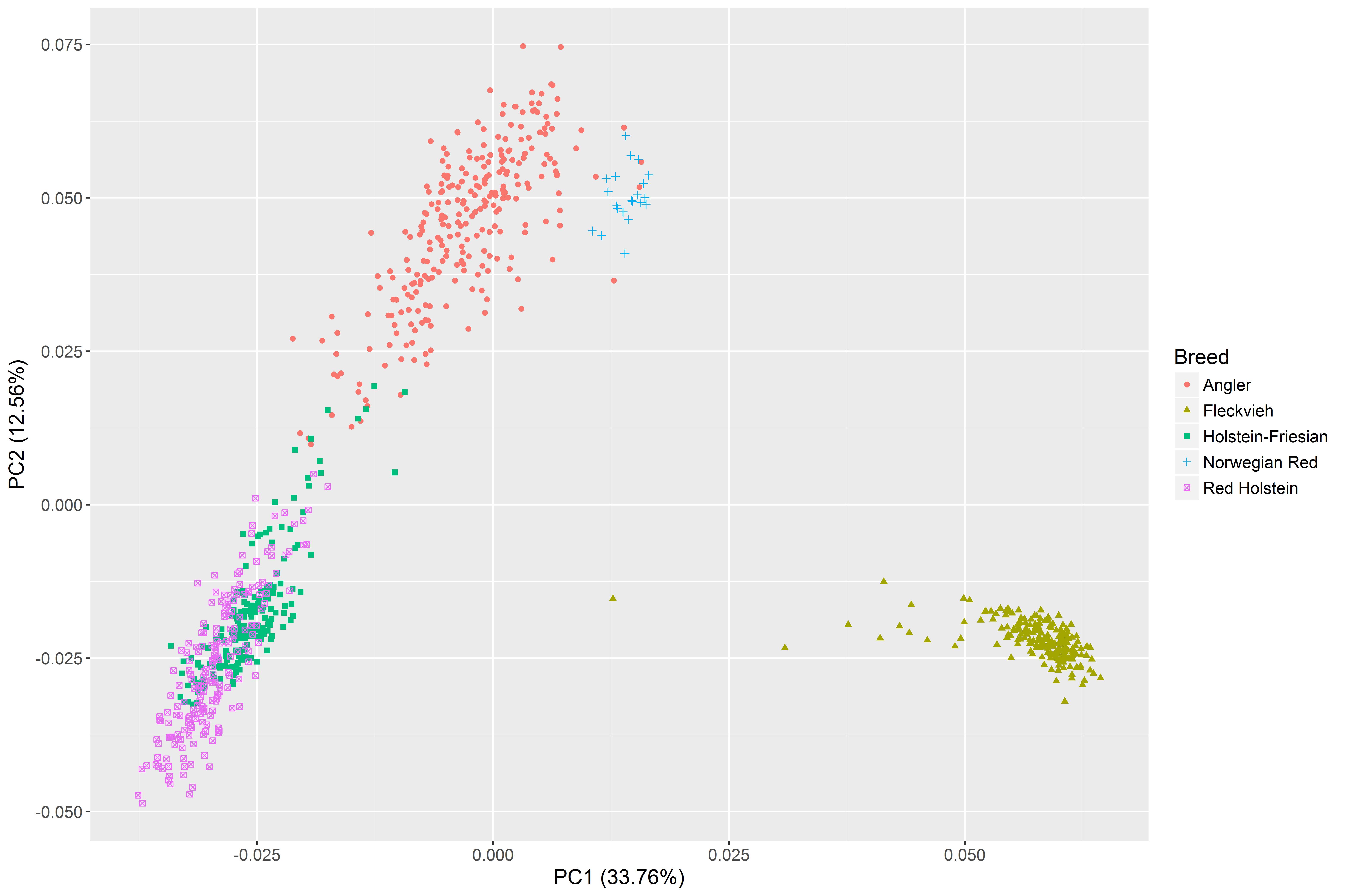

Supplement: Supplementary file 1 [file 4009FigureS1.jpg]

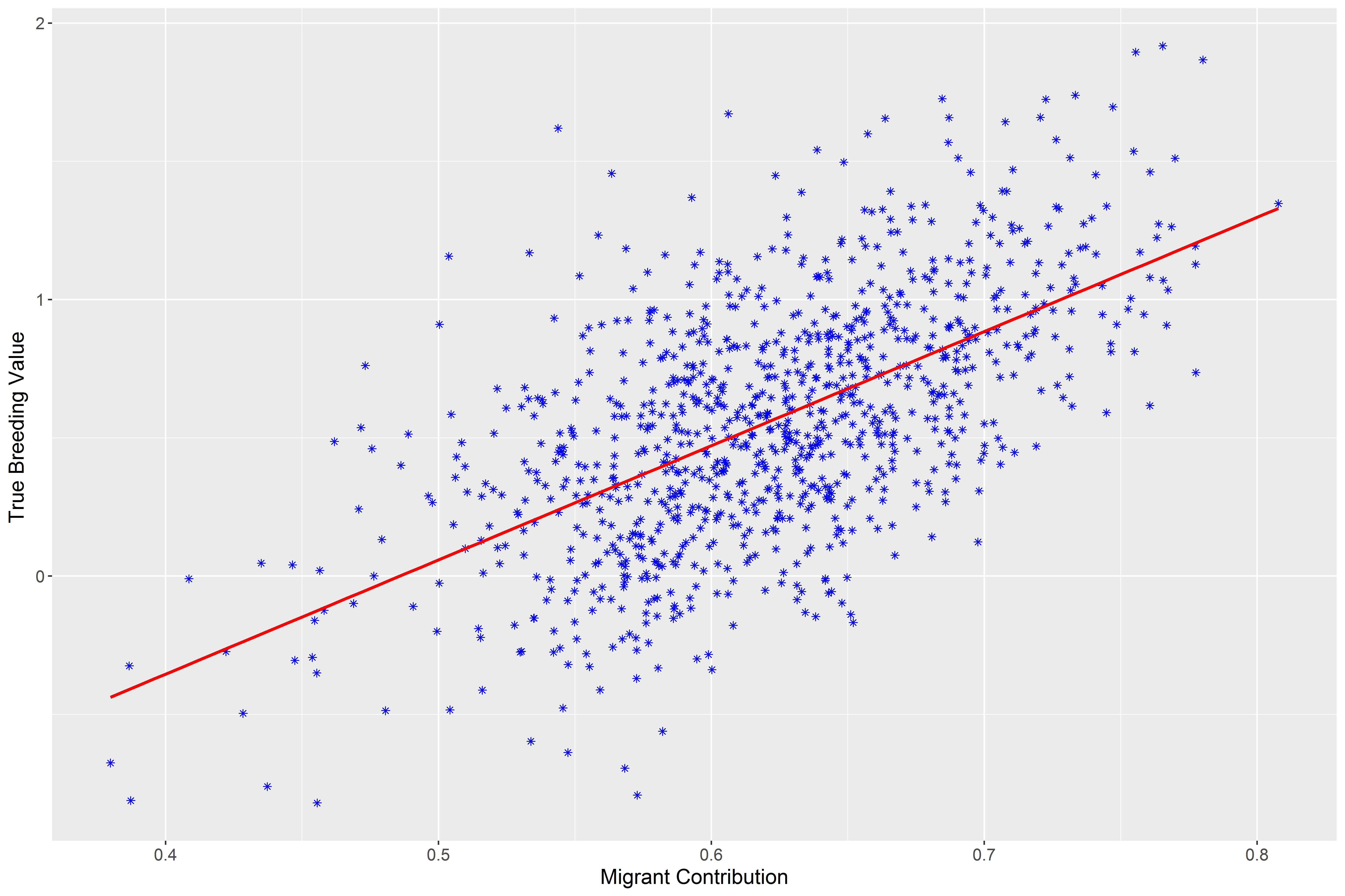

Supplement: Supplementary file 2 [file 4009FigureS2.jpg]

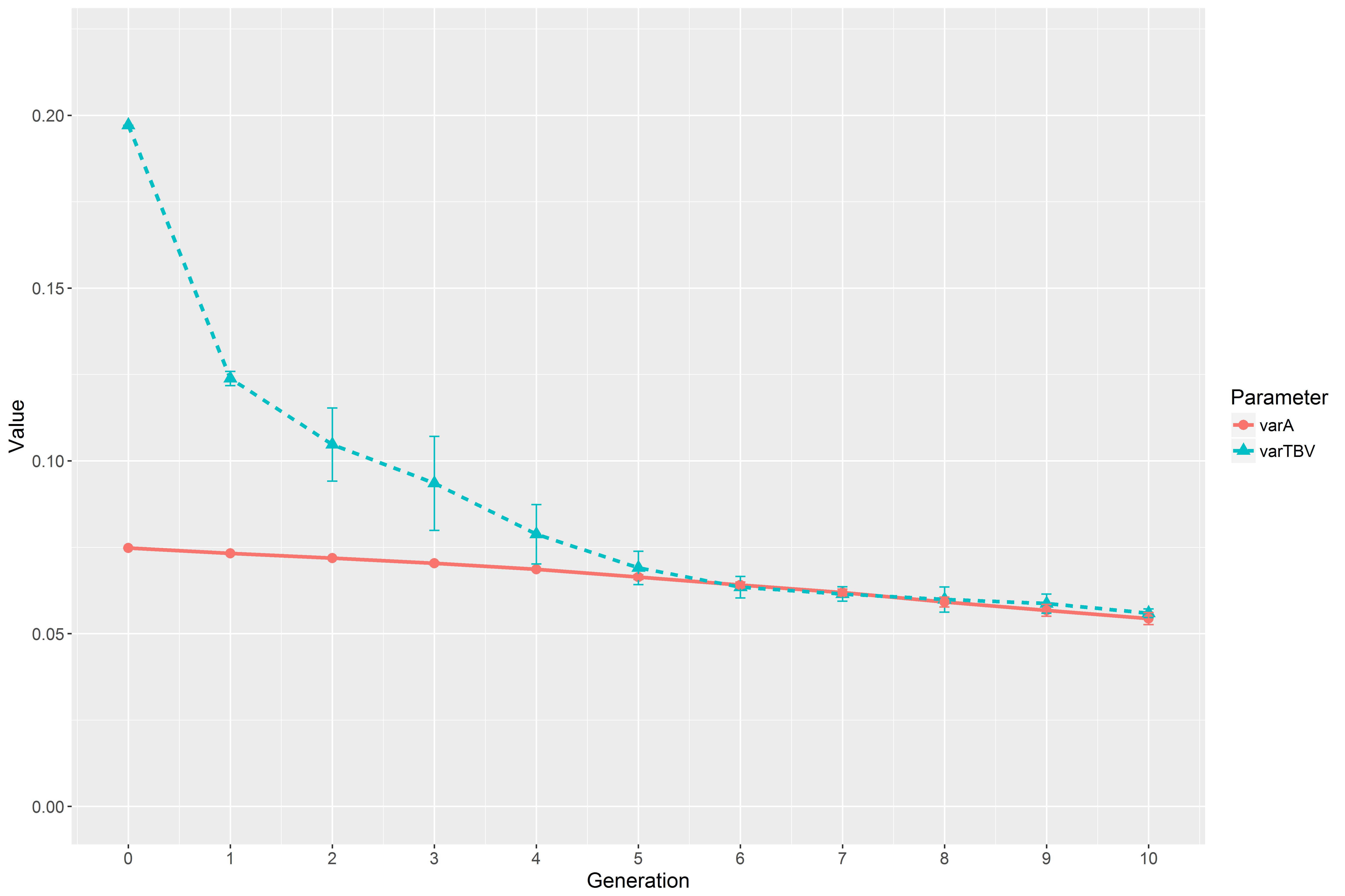

Supplement: Supplementary file 3 [file 4009FigureS3.jpg]
