# Supplementary material for: Long-Term Impact of Optimum Contribution Selection Strategies on Local Livestock Breeds with Historical Introgression Using the Example of German Angler Cattle
Source: G3 (Bethesda). 2017 Oct 31;7(12):4009–18. doi: 10.1534/g3.117.300272 (PMC5714497; doi:10.1534/g3.117.300272)
Supplement: Supplementary file 6 [file 4009FileS3.docx]

Table S1. Constraint settings of each parameter of each generation for specified scenarios.

|  | $\mathbf{ub.}\mathbf{f}_{\mathbf{SEG}}$ | $\mathbf{ub.}\mathbf{f}_{\mathbf{SEG\vert N}}$ | $\mathbf{ub.MC}$ |
| --- | --- | --- | --- |
| $G_{1}$ | 0.053 | 0.065 | 0.603 |
| $G_{2}$ | 0.058 | 0.070 | 0.585 |
| $G_{3}$ | 0.063 | 0.075 | 0.567 |
| $G_{4}$ | 0.067 | 0.079 | 0.550 |
| $G_{5}$ | 0.072 | 0.084 | 0.534 |
| $G_{6}$ | 0.077 | 0.088 | 0.518 |
| $G_{7}$ | 0.081 | 0.093 | 0.502 |
| $G_{8}$ | 0.086 | 0.097 | 0.487 |
| $G_{9}$ | 0.090 | 0.102 | 0.472 |
| $G_{10}$ | 0.095 | 0.106 | 0.458 |
| Applied in Scenario | *OCS-I, OCS-II, OCS-III* | *OCS-II, OCS-III* | *OCS-III* |

Table S2. Descriptive statistics of estimated breeding values (EBV) achieved in each generation for each selection scenario.

| **Scenario** | ***REF*** | | ***TS*** | | ***OCS-I*** | | ***OCS-II*** | | ***OCS-III*** | |
| --- | --- | --- | --- | --- | --- | --- | --- | --- | --- | --- |
|  | **Mean** | **SD** | **Mean** | **SD** | **Mean** | **SD** | **Mean** | **SD** | **Mean** | **SD** |
| $G_{0}$ | 0.561 | 0.000 | 0.561 | 0.000 | 0.561 | 0.000 | 0.561 | 0.000 | 0.561 | 0.000 |
| $G_{1}$ | 0.554 | 0.014 | 0.963 | 0.008 | 0.954 | 0.008 | 0.935 | 0.007 | 0.878 | 0.009 |
| $G_{2}$ | 0.557 | 0.010 | 1.270 | 0.028 | 1.261 | 0.039 | 1.194 | 0.018 | 1.057 | 0.021 |
| $G_{3}$ | 0.555 | 0.010 | 1.531 | 0.039 | 1.491 | 0.031 | 1.396 | 0.014 | 1.158 | 0.043 |
| $G_{4}$ | 0.557 | 0.014 | 1.772 | 0.045 | 1.718 | 0.022 | 1.591 | 0.024 | 1.272 | 0.052 |
| $G_{5}$ | 0.560 | 0.012 | 2.000 | 0.046 | 1.946 | 0.022 | 1.796 | 0.034 | 1.385 | 0.064 |
| $G_{6}$ | 0.560 | 0.013 | 2.219 | 0.045 | 2.153 | 0.034 | 1.992 | 0.038 | 1.472 | 0.075 |
| $G_{7}$ | 0.555 | 0.016 | 2.421 | 0.058 | 2.363 | 0.034 | 2.183 | 0.040 | 1.569 | 0.090 |
| $G_{8}$ | 0.561 | 0.017 | 2.627 | 0.063 | 2.557 | 0.023 | 2.382 | 0.050 | 1.655 | 0.105 |
| $G_{9}$ | 0.559 | 0.017 | 2.815 | 0.055 | 2.747 | 0.030 | 2.568 | 0.049 | 1.745 | 0.103 |
| $G_{10}$ | 0.558 | 0.020 | 3.002 | 0.062 | 2.915 | 0.026 | 2.757 | 0.059 | 1.825 | 0.106 |

Table S3. Descriptive statistics of migrant contribution (MC) achieved in each generation for each scenario.

| **Scenario** | | ***REF*** | | | ***TS*** | | | ***OCS-I*** | | | ***OCS-II*** | | | ***OCS-III*** | |
| --- | --- | --- | --- | --- | --- | --- | --- | --- | --- | --- | --- | --- | --- | --- | --- |
|  | **Mean** | | **SD** | **Mean** | | **SD** | **Mean** | | **SD** | **Mean** | | **SD** | **Mean** | | **SD** |
| $G_{0}$ | 0.622 | | 0.000 | 0.622 | | 0.000 | 0.622 | | 0.000 | 0.622 | | 0.000 | 0.622 | | 0.000 |
| $G_{1}$ | 0.618 | | 0.001 | 0.652 | | <0.001 | 0.634 | | <0.001 | 0.618 | | <0.001 | 0.600 | | 0.001 |
| $G_{2}$ | 0.615 | | 0.001 | 0.667 | | 0.004 | 0.643 | | 0.004 | 0.618 | | 0.001 | 0.582 | | <0.001 |
| $G_{3}$ | 0.612 | | 0.001 | 0.674 | | 0.004 | 0.647 | | 0.005 | 0.618 | | <0.001 | 0.564 | | 0.001 |
| $G_{4}$ | 0.609 | | 0.002 | 0.676 | | 0.006 | 0.647 | | 0.007 | 0.618 | | <0.001 | 0.547 | | 0.001 |
| $G_{5}$ | 0.605 | | 0.002 | 0.678 | | 0.006 | 0.646 | | 0.008 | 0.618 | | 0.001 | 0.530 | | 0.001 |
| $G_{6}$ | 0.602 | | 0.002 | 0.678 | | 0.006 | 0.645 | | 0.009 | 0.618 | | <0.001 | 0.514 | | <0.001 |
| $G_{7}$ | 0.598 | | 0.002 | 0.681 | | 0.004 | 0.644 | | 0.009 | 0.618 | | <0.001 | 0.499 | | <0.001 |
| $G_{8}$ | 0.595 | | 0.002 | 0.680 | | 0.006 | 0.641 | | 0.009 | 0.617 | | 0.002 | 0.484 | | <0.001 |
| $G_{9}$ | 0.591 | | 0.002 | 0.680 | | 0.004 | 0.639 | | 0.008 | 0.617 | | 0.001 | 0.469 | | <0.001 |
| $G_{10}$ | 0.587 | | 0.002 | 0.679 | | 0.005 | 0.638 | | 0.008 | 0.617 | | 0.001 | 0.455 | | <0.001 |

Table S4. Descriptive statistics of kinship $f_{\mathrm{SEG}}$ achieved in each generation for each scenario.

| **Scenario** | | ***REF*** | | | ***TS*** | | | ***OCS-I*** | | | ***OCS-II*** | | | ***OCS-III*** | |
| --- | --- | --- | --- | --- | --- | --- | --- | --- | --- | --- | --- | --- | --- | --- | --- |
|  | **Mean** | | **SD** | **Mean** | | **SD** | **Mean** | | **SD** | **Mean** | | **SD** | **Mean** | | **SD** |
| $G_{0}$ | 0.048 | | 0.000 | 0.048 | | 0.000 | 0.048 | | 0.000 | 0.048 | | 0.000 | 0.048 | | 0.000 |
| $G_{1}$ | 0.048 | | <0.001 | 0.055 | | <0.001 | 0.053 | | <0.001 | 0.052 | | <0.001 | 0.050 | | <0.001 |
| $G_{2}$ | 0.048 | | <0.001 | 0.063 | | 0.001 | 0.058 | | <0.001 | 0.056 | | <0.001 | 0.052 | | <0.001 |
| $G_{3}$ | 0.048 | | <0.001 | 0.070 | | 0.001 | 0.062 | | <0.001 | 0.058 | | 0.001 | 0.054 | | <0.001 |
| $G_{4}$ | 0.047 | | <0.001 | 0.078 | | 0.001 | 0.067 | | <0.001 | 0.062 | | 0.001 | 0.056 | | 0.001 |
| $G_{5}$ | 0.047 | | <0.001 | 0.085 | | 0.001 | 0.072 | | <0.001 | 0.065 | | 0.001 | 0.059 | | 0.001 |
| $G_{6}$ | 0.046 | | <0.001 | 0.092 | | 0.002 | 0.076 | | <0.001 | 0.069 | | 0.001 | 0.061 | | <0.001 |
| $G_{7}$ | 0.045 | | <0.001 | 0.097 | | 0.003 | 0.081 | | <0.001 | 0.073 | | 0.001 | 0.064 | | <0.001 |
| $G_{8}$ | 0.045 | | <0.001 | 0.104 | | 0.004 | 0.085 | | <0.001 | 0.077 | | 0.001 | 0.067 | | <0.001 |
| $G_{9}$ | 0.045 | | <0.001 | 0.109 | | 0.005 | 0.090 | | <0.001 | 0.081 | | 0.001 | 0.070 | | 0.001 |
| $G_{10}$ | 0.044 | | <0.001 | 0.115 | | 0.005 | 0.094 | | <0.001 | 0.085 | | 0.001 | 0.073 | | 0.001 |

Table S5. Descriptive statistics of kinship at native alleles $f_{SEG|N}$ achieved in each generation for each scenario.

| **Scenario** | | ***REF*** | | | ***TS*** | | | ***OCS-I*** | | | ***OCS-II*** | | | ***OCS-III*** | |
| --- | --- | --- | --- | --- | --- | --- | --- | --- | --- | --- | --- | --- | --- | --- | --- |
|  | **Mean** | | **SD** | **Mean** | | **SD** | **Mean** | | **SD** | **Mean** | | **SD** | **Mean** | | **SD** |
| $G_{0}$ | 0.061 | | <0.000 | 0.061 | | 0.000 | 0.061 | | 0.000 | 0.061 | | 0.000 | 0.061 | | 0.000 |
| $G_{1}$ | 0.061 | | <0.001 | 0.066 | | <0.001 | 0.066 | | <0.001 | 0.064 | | <0.001 | 0.064 | | <0.001 |
| $G_{2}$ | 0.059 | | <0.001 | 0.077 | | 0.001 | 0.073 | | 0.002 | 0.069 | | <0.001 | 0.069 | | <0.001 |
| $G_{3}$ | 0.058 | | <0.001 | 0.091 | | 0.001 | 0.081 | | 0.002 | 0.073 | | <0.001 | 0.073 | | <0.001 |
| $G_{4}$ | 0.056 | | <0.001 | 0.104 | | 0.002 | 0.089 | | 0.001 | 0.078 | | <0.001 | 0.078 | | <0.001 |
| $G_{5}$ | 0.055 | | <0.001 | 0.116 | | 0.003 | 0.099 | | 0.002 | 0.082 | | <0.001 | 0.082 | | 0.001 |
| $G_{6}$ | 0.054 | | <0.001 | 0.128 | | 0.006 | 0.108 | | 0.001 | 0.086 | | <0.001 | 0.087 | | <0.001 |
| $G_{7}$ | 0.051 | | <0.001 | 0.136 | | 0.007 | 0.115 | | 0.001 | 0.091 | | <0.001 | 0.091 | | <0.001 |
| $G_{8}$ | 0.050 | | <0.001 | 0.144 | | 0.008 | 0.123 | | 0.001 | 0.095 | | <0.001 | 0.096 | | <0.001 |
| $G_{9}$ | 0.049 | | 0.001 | 0.151 | | 0.009 | 0.129 | | 0.003 | 0.099 | | <0.001 | 0.100 | | <0.001 |
| $G_{10}$ | 0.048 | | 0.001 | 0.157 | | 0.009 | 0.136 | | 0.004 | 0.104 | | <0.001 | 0.104 | | <0.001 |

Table S6. Descriptive statistics of observed heterozygosity ($H_{O}$) achieved in each generation for each scenario.

| **Scenario** | | ***REF*** | | | ***TS*** | | | ***OCS-I*** | | | ***OCS-II*** | | | ***OCS-III*** | |
| --- | --- | --- | --- | --- | --- | --- | --- | --- | --- | --- | --- | --- | --- | --- | --- |
|  | **Mean** | | **SD** | **Mean** | | **SD** | **Mean** | | **SD** | **Mean** | | **SD** | **Mean** | | **SD** |
| $G_{0}$ | 0.367 | | 0.000 | 0.367 | | 0.000 | 0.367 | | 0.000 | 0.367 | | 0.000 | 0.367 | | 0.000 |
| $G_{1}$ | 0.367 | | <0.001 | 0.365 | | 0.001 | 0.367 | | <0.001 | 0.367 | | <0.001 | 0.367 | | <0.001 |
| $G_{2}$ | 0.365 | | <0.001 | 0.363 | | <0.001 | 0.364 | | 0.001 | 0.364 | | <0.001 | 0.364 | | 0.001 |
| $G_{3}$ | 0.365 | | <0.001 | 0.360 | | <0.001 | 0.362 | | 0.001 | 0.363 | | 0.001 | 0.362 | | 0.001 |
| $G_{4}$ | 0.365 | | <0.001 | 0.357 | | 0.001 | 0.360 | | 0.001 | 0.361 | | 0.001 | 0.361 | | <0.001 |
| $G_{5}$ | 0.365 | | <0.001 | 0.355 | | 0.001 | 0.359 | | 0.001 | 0.360 | | 0.001 | 0.360 | | <0.001 |
| $G_{6}$ | 0.364 | | <0.001 | 0.352 | | 0.001 | 0.357 | | 0.001 | 0.358 | | <0.001 | 0.359 | | <0.001 |
| $G_{7}$ | 0.364 | | <0.001 | 0.351 | | 0.002 | 0.356 | | 0.001 | 0.357 | | <0.001 | 0.358 | | 0.001 |
| $G_{8}$ | 0.364 | | <0.001 | 0.349 | | 0.002 | 0.354 | | 0.001 | 0.355 | | 0.001 | 0.357 | | 0.001 |
| $G_{9}$ | 0.364 | | <0.001 | 0.347 | | 0.003 | 0.352 | | 0.001 | 0.354 | | 0.001 | 0.356 | | <0.001 |
| $G_{10}$ | 0.364 | | <0.001 | 0.346 | | 0.002 | 0.351 | | 0.001 | 0.353 | | 0.001 | 0.355 | | 0.001 |

Table S7. Descriptive statistics of the variance of true breeding values ($\sigma_{\mathrm{TBV}}^{2}$) achieved in each generation for each scenario.

| **Scenario** | ***REF*** | | ***TS*** | | ***OCS-I*** | | ***OCS-II*** | | ***OCS-III*** | |
| --- | --- | --- | --- | --- | --- | --- | --- | --- | --- | --- |
|  | **Mean** | **SD** | **Mean** | **SD** | **Mean** | **SD** | **Mean** | **SD** | **Mean** | **SD** |
| $G_{0}$ | 0.197 | 0.000 | 0.197 | 0.000 | 0.197 | 0.000 | 0.197 | 0.000 | 0.197 | 0.000 |
| $G_{1}$ | 0.197 | 0.000 | 0.112 | 0.005 | 0.120 | 0.002 | 0.124 | 0.002 | 0.121 | 0.003 |
| $G_{2}$ | 0.150 | 0.008 | 0.085 | 0.004 | 0.092 | 0.010 | 0.105 | 0.011 | 0.110 | 0.009 |
| $G_{3}$ | 0.124 | 0.005 | 0.075 | 0.003 | 0.082 | 0.012 | 0.094 | 0.014 | 0.095 | 0.007 |
| $G_{4}$ | 0.111 | 0.004 | 0.068 | 0.003 | 0.072 | 0.009 | 0.079 | 0.009 | 0.082 | 0.005 |
| $G_{5}$ | 0.102 | 0.006 | 0.062 | 0.003 | 0.074 | 0.011 | 0.069 | 0.005 | 0.078 | 0.010 |
| $G_{6}$ | 0.093 | 0.002 | 0.056 | 0.004 | 0.064 | 0.005 | 0.064 | 0.003 | 0.079 | 0.009 |
| $G_{7}$ | 0.093 | 0.002 | 0.055 | 0.002 | 0.059 | 0.005 | 0.062 | 0.002 | 0.075 | 0.004 |
| $G_{8}$ | 0.089 | 0.005 | 0.052 | 0.003 | 0.055 | 0.003 | 0.060 | 0.004 | 0.075 | 0.004 |
| $G_{9}$ | 0.091 | 0.003 | 0.049 | 0.002 | 0.052 | 0.004 | 0.059 | 0.003 | 0.071 | 0.005 |
| $G_{10}$ | 0.091 | 0.005 | 0.044 | 0.002 | 0.049 | 0.002 | 0.056 | 0.001 | 0.065 | 0.003 |

Table S8. Descriptive statistics of the average genic variance ($\sigma_{A}^{2}$) achieved in each generation for each scenario.

| **Scenario** | | ***REF*** | | | ***TS*** | | | ***OCS-I*** | | | ***OCS-II*** | | | ***OCS-III*** | |
| --- | --- | --- | --- | --- | --- | --- | --- | --- | --- | --- | --- | --- | --- | --- | --- |
|  | **Mean** | | **SD** | **Mean** | | **SD** | **Mean** | | **SD** | **Mean** | | **SD** | **Mean** | | **SD** |
| $G_{0}$ | 0.075 | | 0.000 | 0.075 | | 0.000 | 0.075 | | 0.000 | 0.075 | | 0.000 | 0.075 | | 0.000 |
| $G_{1}$ | 0.075 | | <0.001 | 0.073 | | <0.001 | 0.073 | | <0.001 | 0.073 | | <0.001 | 0.073 | | <0.001 |
| $G_{2}$ | 0.075 | | <0.001 | 0.072 | | <0.001 | 0.072 | | <0.001 | 0.072 | | <0.001 | 0.073 | | <0.001 |
| $G_{3}$ | 0.075 | | <0.001 | 0.070 | | <0.001 | 0.070 | | <0.001 | 0.070 | | <0.001 | 0.071 | | <0.001 |
| $G_{4}$ | 0.075 | | <0.001 | 0.067 | | <0.001 | 0.068 | | 0.001 | 0.069 | | <0.001 | 0.070 | | <0.001 |
| $G_{5}$ | 0.075 | | <0.001 | 0.064 | | 0.001 | 0.065 | | 0.001 | 0.066 | | 0.001 | 0.069 | | 0.001 |
| $G_{6}$ | 0.075 | | <0.001 | 0.061 | | 0.001 | 0.063 | | 0.002 | 0.064 | | 0.001 | 0.068 | | 0.001 |
| $G_{7}$ | 0.075 | | <0.001 | 0.058 | | 0.001 | 0.060 | | 0.002 | 0.062 | | 0.001 | 0.067 | | 0.001 |
| $G_{8}$ | 0.075 | | <0.001 | 0.055 | | 0.001 | 0.057 | | 0.001 | 0.059 | | 0.002 | 0.066 | | 0.002 |
| $G_{9}$ | 0.075 | | <0.001 | 0.052 | | 0.001 | 0.054 | | 0.002 | 0.057 | | 0.002 | 0.065 | | 0.002 |
| $G_{10}$ | 0.075 | | <0.001 | 0.049 | | 0.001 | 0.052 | | 0.002 | 0.054 | | 0.002 | 0.063 | | 0.002 |
